# Aphid galls as a double food source for woodpeckers

**DOI:** 10.1002/ece3.8994

**Published:** 2022-06-02

**Authors:** Tiziano Londei

**Affiliations:** ^1^ Independent Researcher Milan Italy

**Keywords:** gall‐contained aphids and honeydew, gall‐opening birds, unusual/overlooked feeding behavior

## Abstract

An adult female and a young great spotted woodpecker *Dendrocopos major* were found exploiting galls of the elm balloon‐gall aphid *Eriosoma lanuginosum* by eating both the insects and their honeydew. Literature offers sparse and indirect information to explain this behavior. Taking both aphids and honeydew from within galls is reported here for the first time for a bird species. Only one other vertebrate, the Eurasian red squirrel *Sciurus vulgaris*, would show similar behavior. In contrast with the seeming rarity of such a feeding way, likely advantages in the double food source suggest it may have been overlooked.

## Graphical abstract

Great spotted woodpeckers were found exploiting galls of the elm balloon‐gall aphid by eating both the insects and their honeydew, a behavior reported here for the first time for a bird species. In contrast with the seeming rarity of such a feeding way, likely advantages in the double food source suggest it may have been overlooked.

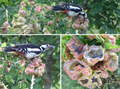

## 1 INTRODUCTION

**FIGURE 1:**
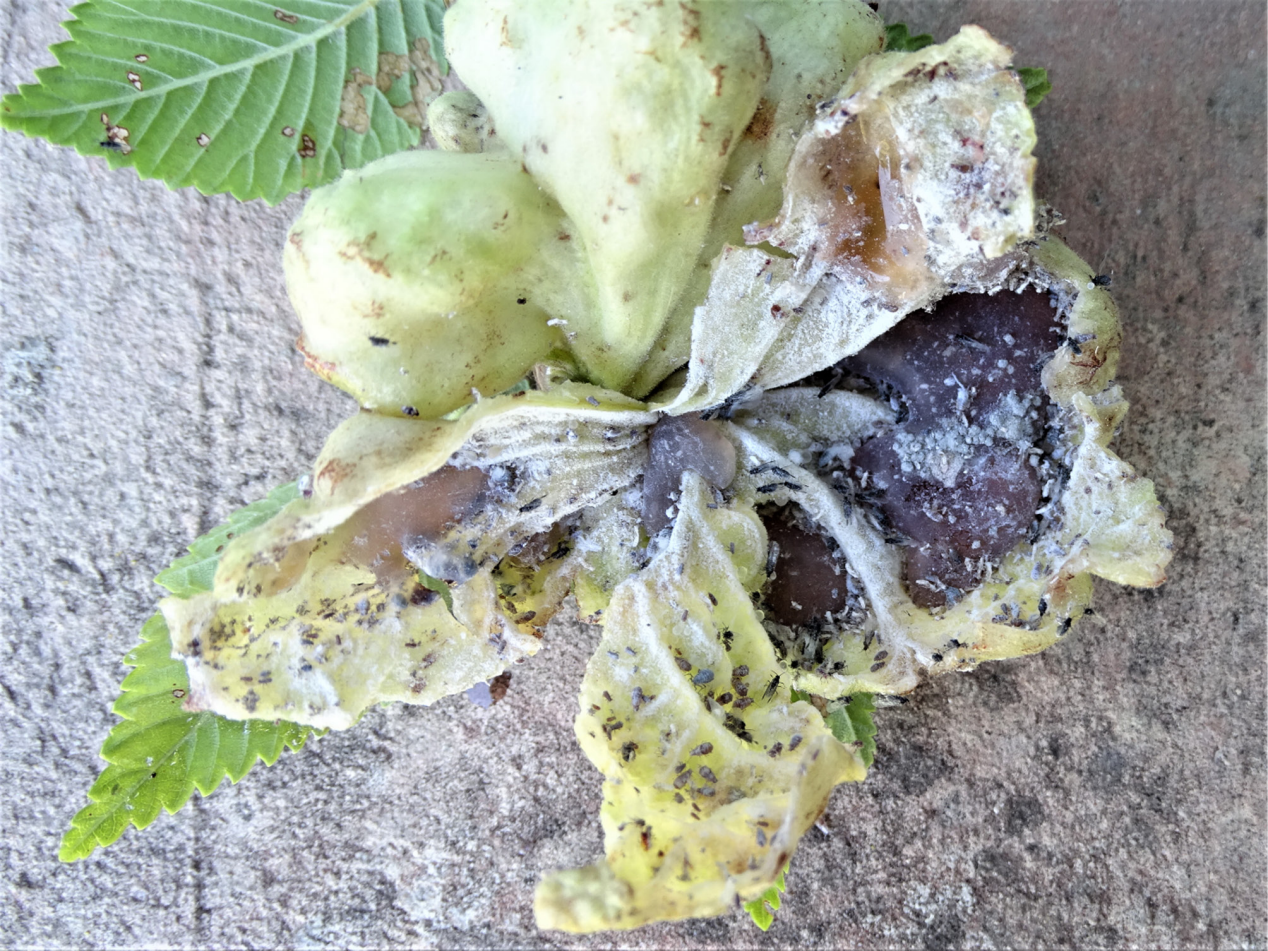
A gall of *Eriosoma lanuginosum* on *Ulmus minor*, opened by the author when it was still unfissured but already full of winged, in addition to unwinged, aphids. Also note the abundant honeydew in coalesced sediment

The elm balloon‐gall aphid *Eriosoma lanuginosum* induces *Ulmus* trees to produce large bloated‐leaf galls and several generations of wingless aphids live inside the growing gall until it fissures, permitting winged aphids to move to a different host (Blackman & Eastop, 2013). Honeydew, the aphid sugary excrement, is produced in droplets which in mature galls coalesce in massive sediment, and the aphids avoid being entrapped in it by producing a powdery hydrophobic wax which coats every element inside the gall (Figure 1). While many invertebrates are known as predators of galling insects (e.g., Stone & Schönrogge, 2003), few bird species have been identified for this role (Burstein & Wool, 1992; László et al., 2014; Moeller & Thogerson, 1978; Schlichter, 1978; Schönrogge et al., 2013; Sunose, 1980). Most of these birds are passerines, chiefly tits, but some are woodpeckers (László et al., 2014; Moeller & Thogerson, 1978; Schönrogge et al., 2013). Compared to smaller woodpeckers (see especially the downy woodpecker *Dryobates pubescens*), the great spotted woodpecker *Dendrocopos major* seems very rarely observed while eating from galls: I could read only one report in this respect, of a single bird extracting larvae of the large cigar gall fly *Lipara lucens* (Kramer, 1917). Although detailed observations made during the subject's feeding activity are scarce, it seems that all the observed bird species were searching in the gall only for its occupants. No bird was reported taking honeydew from within galls, although several bird species do eat honeydew, if freely available (e.g., Latta et al., 2001). Here, I report on the great spotted woodpecker opening galls of the elm balloon‐gall aphid and eating both the aphids and their honeydew.

## 2 RESULTS

**FIGURE 2:**
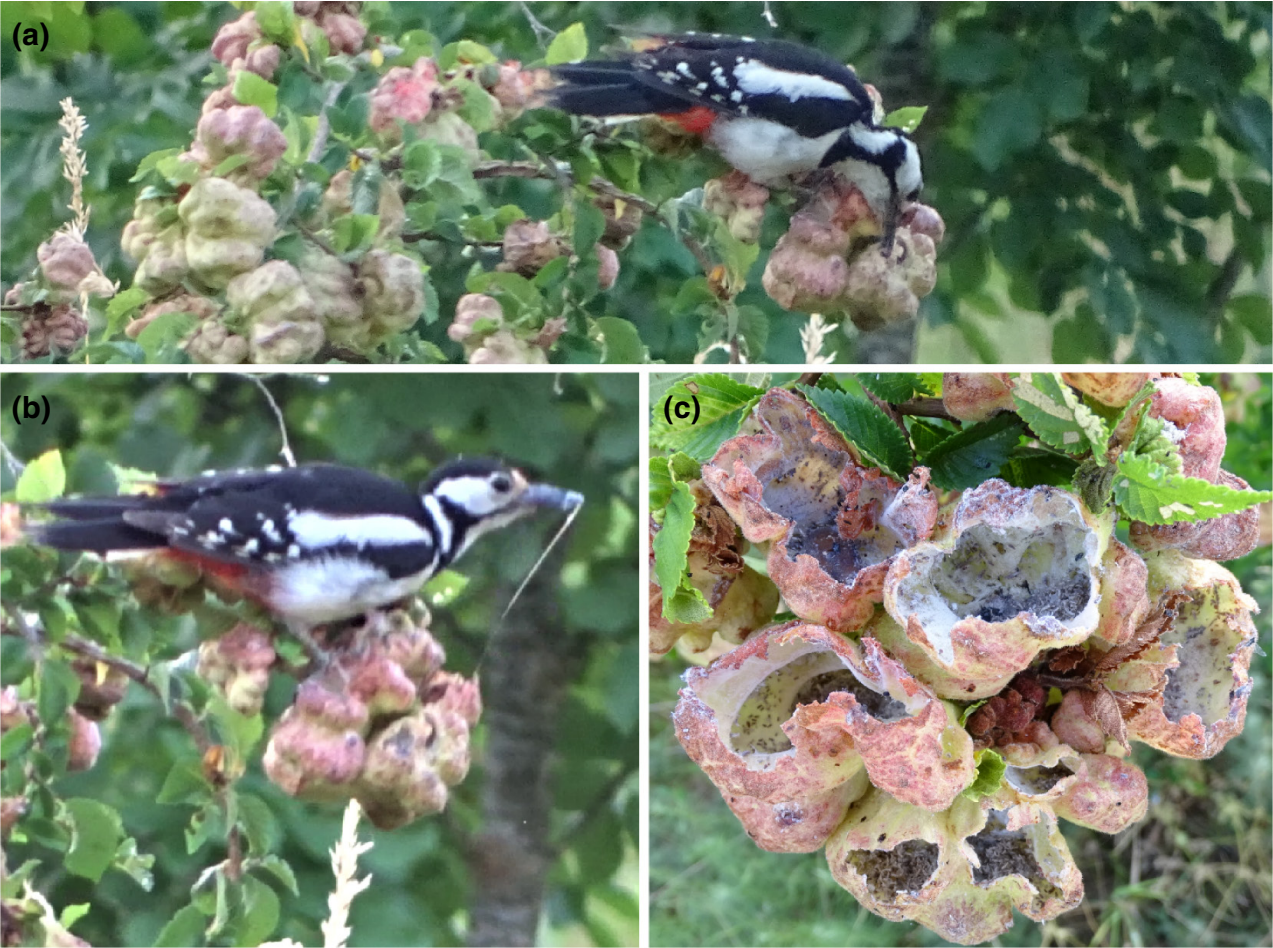
The adult female Great Spotted Woodpecker tearing open a gall (a) and eating honeydew from the interior (b). The same cluster of galls just after the woodpecker's feeding activity (c): note the scarcity of both aphids, exuviae apart, and honeydew left in these galls compared to Figure 1

On June 21, 2021, 7:07 local time, from my country house (N 44°46′ E 9°23′, 300 m asl) near Bobbio (PC), Italy, I noticed an unusual behavior of the female great spotted woodpecker of the pair residing nearby. She flew directly to an exposed cluster of galls in a growth of field elms *Ulmus minor* heavily infested by the elm balloon‐gall aphid and began pecking at the galls. Staying behind the glass of a closed window, I could observe this usually wary bird undisturbed and obtain images of its feeding activity. Of three galls exploited in four minutes, at least one was pierced for the first time during my observation. After tearing off pieces of gall tissue without eating them, the bird first nibbled in the cavity and later probed deeply, so that honeydew remained stuck to its bill (Figure 2). By comparing open and equally mature intact galls for the amount of their content I had confirmation that both aphids and coalesced honeydew were eaten. On the same day, I found galls opened in the same way on two other elms nearby, a sign that this feeding mode had begun some days earlier. From June 21 to June 30, a day when most of the galls had already dehisced, I kept the elm growth under observation, daily in the early morning and often also in other parts of the day. On June 26, 6:20, a young bird came alone and fed on a previously exploited gall. This bird kept feeding for just one minute, and unlike the adult female it ate pieces of gall tissue and did not nibble in the cavity. Subsequent examination showed remnants of thickened honeydew stuck to the cavity, which suggests the dried‐up state of the gall being the cause of the behavioral difference. On June 25, 6:15, and June 29, 7:02, I saw the adult male, without him paying any attention to galls, although clearly searching for insects in elm canopies. The same lack of interest in galls was apparent in other resident birds that I observed foraging in the elm growth almost every day: great tits *Parus major*, Eurasian blue tits *Cyanistes caeruleus*, and Eurasian jays *Garrulus glandarius*. I saw none of these birds contacting the galls, not even those open enough to clearly show their content. Formerly used for subsistence agriculture, but left to natural vegetation since many years, the area around my house offers a rich choice of food to birds with its various species of old, isolated, planted fruit trees interspersed with younger, loose, wild trees.

## 3 DISCUSSION

The great spotted woodpecker would seem the only bird species feeding on both aphids and their honeydew from galls. The Eurasian red squirrel *Sciurus vulgaris* would be the only other vertebrate behaving similarly, when the number of aphids and the amount of honeydew in galls of the elm‐currant aphid *Eriosoma ulmi* are largest (Janiszewska‐Cichocka, 1971 in Urban, 2003), although there is no mention of this occurrence in a recent review of the Eurasian red squirrel's diet (Krauze‐Gryz & Gryz, 2015). Such scarcity of previous reports, as well as the obvious lack of interest in galls on the part of several birds during my observations, would point to rare behavior. Burstein and Wool (1992) proposed for the great tit local discovery of aphid galls as food sources, explaining it with birds having noticed aphids coming out of the galls. Thus, gall‐opening behavior might depend on aphid outbursts: in the present case, a frequent emergence of winged aphids from fissured mature galls may have led a woodpecker to enlarge some fissure to reach the gall interior, thus discovering the double food source. The young bird's behavior, probably a residual activity at a decreasing food source, suggests that interest in galls may have been passed onto offspring. Accordingly, Sunose (1980) observed birds foraging together with their fledglings on galled trees. Schönrogge et al. (2013) found a prevalence of Eurasian blue tits and great tits among gall‐opening birds and saw the tit‐typical accelerating learning mode in the exploitation of new food sources as a possibility worthy of consideration. The contrasting absence of interest in galls I observed in the two tit species suggests to me that woodpeckers were more predisposed to discovery in this case. I considered the possibility that these woodpeckers had at first been attracted to the galled elms as sap‐sucking birds, because elms are among the favorite trees in this respect (Gibbs, 1983). However, in the bark of these elms I did not find the typical “rings” of small holes that woodpeckers make to obtain the sap. As a more likely explanation I propose that the balloon‐like galls of these aphids easily reveal themselves as hollow structures, and, being predisposed to detect prey that burrows into trees, woodpeckers are especially responsive to hidden hollows. Once a gall was exploited by a woodpecker, other birds might find scarce (too sticky?) food in the remaining mixture of aphids and drying honeydew, and thus ignore similar galls. Whatever the origin of this gall‐opening behavior, a mixture of aphids and honeydew might be very profitable food. Great spotted woodpeckers usually forage on freely available aphids during the breeding season (e.g., Rolstad et al., 1995). Concerning phloem sap, Pakkala et al. (2018) judged it a possible, more predictable, energy‐rich, and nutritionally valuable substitute for insects in the diet of breeding woodpeckers. However, they admitted that it was still unclear whether so much sap was used because of its easy availability, plus depleted availability of invertebrates, or because of an actual preference for sap, or a mixture of sap and invertebrates. Honeydew may be better food than phloem sap, as it is physiologically less extreme, with a higher essential/non‐essential amino acid ratio and lower osmotic pressure (Douglas, 2006). Providing that such exploitation of aphid galls by relatively large animals was sufficiently widespread, it might affect aphid populations considerably, because habitat alteration inside a widely opened gall could by itself kill all the wingless aphids still alive after the attacks of the predator. Future study may ascertain whether such behavior is truly rare, being maybe the result of individual discoveries by predisposed animals during aphid outbreaks, or it has been overlooked, likely because of a general lack of attention to opportunistic gall exploitation by vertebrates.

## AUTHOR CONTRIBUTION

**Tiziano Londei:** Conceptualization (lead); Investigation (lead); Validation (lead); Visualization (lead); Writing – original draft (lead); Writing – review & editing (lead).

## CONFLICT OF INTEREST

None declared.

## Data Availability

Data sharing is not applicable to this article because no new data were created or analyzed in this study.
